# Towards the Characterization and Engineering of Bacteriophages in the Gut Microbiome

**DOI:** 10.1128/mSystems.00735-21

**Published:** 2021-08-24

**Authors:** Bryan B. Hsu

**Affiliations:** a Department of Biological Sciences, Virginia Tech, Blacksburg, Virginia, USA

**Keywords:** phage, synthetic biology, gut microbiome

## Abstract

The gut microbiome and its importance to human health are a rapidly evolving area of study. Bacteria often take center stage. However, the composition is much more complex with other microbial members of the gut also playing key roles. Bacteriophages (phages), the viruses that infect bacteria, are an integral component of gut microbiomes and can often be found cocolonizing with their commensal bacterial hosts. Recent studies have shown associations between the composition of resident phage communities and human health and disease, but the mechanisms of these associations remain elusive. My research laboratory is focused on understanding the role of phages in the gut microbiome and exploring their possible therapeutic applications.

## COMMENTARY

Bacteriophages (phages), the viruses of bacteria, are a major component of global ecosystems with a total population estimated to be ∼10^31^ viruses (1). In the human body, the gut is the most abundantly populated region with individual-specific communities of phages that have extensive and often entangled fates with their host bacteria. Most phages can be classified as virulent or temperate. Successful infection by a virulent phage generally leads to cell lysis, the release of progeny, and the continuation of phage propagation. In this case, “virulent” refers to the capability of phage to pursue lytic replication. While temperate phages can similarly pursue a lytic life cycle to produce free virions, they can also remain intracellular in a “dormant” form after infection, often integrating into the bacterial chromosome as a prophage (i.e., lysogeny) until environmental or cellular conditions favor their return to lytic propagation. The consequences of these two life cycles under defined *in vitro* conditions have been well studied, but considerably less is known about *in vivo* conditions. How well do these characteristics of phage propagation and bacterial response in liquid culture extend to more complex and diverse conditions like those found in the gut microbiome? Are there features that can be leveraged to improve our mechanistic understanding of the gut microbiota, and can this new knowledge be used to identify druggable targets? My research laboratory aims to develop experimental strategies that will improve our understanding of the role of phage in microbially rich and diverse communities, such as the gut microbiota, and leverage their properties toward the modification of specific bacteria and their functions in the mammalian gut.

## BACTERIOPHAGES—BACTERIAL GRAZING OR DEVOURING

The role of phages in the mammalian gut is an emergent area of research relevant to diseases including inflammatory bowel disease (2), rheumatoid arthritis (3), colorectal cancer (4), and Clostridioides difficile infections (CDI) (5). A key consideration is whether alterations in the phage community contribute to or result from disease. Fecal microbiota transplantation (FMT) has been instructive in this regard. The process of FMT, whereby the fecal microbiota derived from a healthy donor is administered to a patient, has shown remarkable success in treating recurrent CDI (rCDI) (6). While the mechanisms continue to be investigated, engraftment of donor bacteria is generally believed to be a driving force behind microbiome restoration and improved health outcomes, while sustained phage engraftment has also been associated with patient responsiveness to FMT (5). Even the transplantation of a fecal filtrate (i.e., donor material without bacteria) reduced disease symptoms in all rCDI patients (*n* = 5), with sustained donor phage engraftment in the one recipient that was further characterized (7). The undefined and variable nature of donor material used in FMT makes it difficult to draw robust conclusions regarding the potential mechanisms by which donor phage can alter resident microbiota, but their association with remission of rCDI warrants additional investigation.

**FIG 1:**
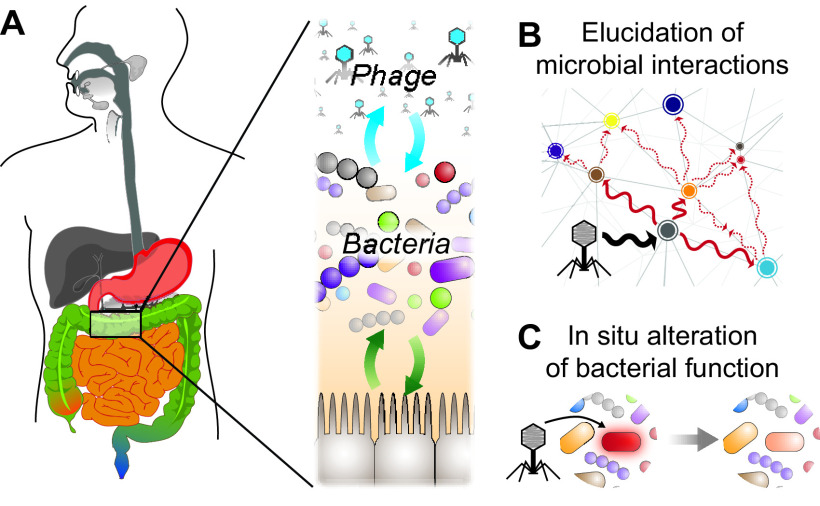
The human gut microbiome is composed of an individual-specific community of phage and bacteria (A). Judicious application of phage in a rich and diverse community like the mammalian gut offers opportunities to perturb specific subsets of bacterial species (B) and introduce or modulate their function at the genetic level (C).

While phage have exquisite specificity for bacteria, often to the species and sometimes to the strain level, they may also have a more expansive impact on polymicrobial communities. For example, phage can decimate a bacterial species in liquid culture, leading to the emergence of a resistant strain that subsequently dominates. While the decreased fitness associated with phage resistance (i.e., reduced expression of a transporter that also serves as a phage receptor) may be inconsequential in monoculture, the presence of a competitor can limit the expansion of this phage-resistant mutant (8). In gnotobiotic mice colonized by a consortium of 10 human gut commensals, we found that the knockdown of specific bacteria by lytic phage altered the overall composition of the microbiota, i.e., those not directly targeted by phage, and the gut metabolome (9). This alteration in the microbiota was largely recapitulated in another set of mice where each bacterial species was omitted, suggesting that the knockdown of a specific species modulates cooperative and competitive interbacterial interactions within the gut. A recent gnotobiotic mouse study examining lytic phage in a consortium of murine commensals did not observe such cascading effects when targeting Escherichia coli (10). These and other recent findings show that much remains to be explored about the mechanisms of interaction between phage and bacteria and the extent of the impact on the mammalian host (Fig. 1A). The specificity of phage for bacterial hosts and their potential for amplified or muted effect in a gut microbiome are an important area of exploration.

## FROM SAWS TO SCALPELS

The microbial specificity of phage makes it a unique and potentially deployable tool in complex polymicrobial ecosystems. Producing targeted changes in the commensal gut microbiota, whether to mechanistically explore microbial interactions or to therapeutically leverage such effects, is challenging due in part to the dearth of precision tools. Antibiotics lack the precision to isolate and track the effects of individual bacterial species. However, virulent phages have the capability of such applications. This approach is under way in phage therapy, where a virulent phage or cocktail of phages can be deployed against a bacterial infection. Targeted phage also provide opportunities to apply such strategies to probe or modulate commensal species in the gut.

My research group is exploring the use of virulent phages to probe the causal nature of bacteria within the mammalian gut. The ability to precisely modulate the colonization of bacteria within a diverse consortium is a distinct advantage for phages. By probing the members of these microbial networks, we have the opportunity to reveal competitive and cooperative interactions within the context of the mammalian host (Fig. 1B). Furthermore, we are interested in the extent to which the resident phage community, both virulent and temperate, impacts the ecological properties of the gut microbiome. Exploration of these areas will provide insights into the role of phage in the gut microbiome.

## ERADICATION OR REHABILITATION

Temperate phages facilitate a mechanism of horizontal gene transfer (HGT) by which bacteria can rapidly expand their genetic diversity. Genomically integrated prophages are abundant in the human gut (11) and can confer fitness on their bacterial hosts (12). We anticipate that it is possible to leverage the advantages of HGT by temperate phage as a means for widespread dissemination of programmable genetic functions. In streptomycin-treated mouse models colonized with E. coli MG1655, we found that lambda phage lysogenizes a substantial fraction of this population in the gut (13), indicating the possibility of its use for delivering heterologous functions to gut bacteria. We have leveraged this strategy for bacterial modification by engineering lambda phage with a repressor of Shiga toxin to inhibit toxin expression in toxin-producing E. coli *in vitro* and in a mouse model of enteric infection (13). We have also engineered lambda phage to express the programmable repressor dCas9 and showed that it stably represses gene expression *in vitro* and *in vivo* (14).

Increasing antimicrobial drug resistance among pathogenic bacteria is sapping the potency of our life-saving medicines. Using engineered temperate phages to neutralize virulence offers an alternative strategy for addressing bacterial infections. My research group is interested in exploring the potential applications of engineered temperate phages toward the modulation of bacterial expression that is not limited to virulence factors but could include the repression or introduction of other functions (Fig. 1C).

## OUTLOOK

Phages are a significant component of the gut microbiome, but their specific role and the extent of their influence remain an opportunity for greater study. Ultimately, leveraging a greater understanding of the gut microbiome will improve human health. Similar to bacteria, the elucidation of the role of phages, particularly those that are resident in the gut and coexist with their bacterial hosts, could provide key information about microbial networks and identify potentially druggable targets. This area of microbiome research is rapidly expanding, and characterizing the role of phages holds great potential.

## References

[1] Suttle CA. 2005. Viruses in the sea. Nature 437:356–361. doi:10.1038/nature04160. 16163346

[2] Norman JM, Handley SA, Baldridge MT, Droit L, Liu CY, Keller BC, Kambal A, Monaco CL, Zhao G, Fleshner P, Stappenbeck TS, McGovern DPB, Keshavarzian A, Mutlu EA, Sauk J, Gevers D, Xavier RJ, Wang D, Parkes M, Virgin HW. 2015. Disease-specific alterations in the enteric virome in inflammatory bowel disease. Cell 160:447–460. doi:10.1016/j.cell.2015.01.002. 25619688 PMC4312520

[3] Mangalea MR, Paez-Espino D, Kieft K, Chatterjee A, Chriswell ME, Seifert JA, Feser ML, Demoruelle MK, Sakatos A, Anantharaman K, Deane KD, Kuhn KA, Holers VM, Duerkop BA. 2021. Individuals at risk for rheumatoid arthritis harbor differential intestinal bacteriophage communities with distinct metabolic potential. Cell Host Microbe 29:726–739.e5. doi:10.1016/j.chom.2021.03.020. 33957082 PMC8186507

[4] Nakatsu G, Zhou H, Wu WKK, Wong SH, Coker OO, Dai Z, Li X, Szeto C-H, Sugimura N, Lam TY-T, Yu AC-S, Wang X, Chen Z, Wong MC-S, Ng SC, Chan MTV, Chan PKS, Chan FKL, Sung JJ-Y, Yu J. 2018. Alterations in enteric virome are associated with colorectal cancer and survival outcomes. Gastroenterology 155:529–541.e5. doi:10.1053/j.gastro.2018.04.018. 29689266

[5] Zuo T, Wong SH, Lam K, Lui R, Cheung K, Tang W, Ching JYL, Chan PKS, Chan MCW, Wu JCY, Chan FKL, Yu J, Sung JJY, Ng SC. 2018. Bacteriophage transfer during faecal microbiota transplantation in *Clostridium difficile* infection is associated with treatment outcome. Gut 67:634–643. doi:10.1136/gutjnl-2017-313952. 28539351 PMC5868238

[6] van Nood E, Vrieze A, Nieuwdorp M, Fuentes S, Zoetendal EG, de Vos WM, Visser CE, Kuijper EJ, Bartelsman JFWM, Tijssen JGP, Speelman P, Dijkgraaf MGW, Keller JJ. 2013. Duodenal infusion of donor feces for recurrent Clostridium difficile. N Engl J Med 368:407–415. doi:10.1056/NEJMoa1205037. 23323867

[7] Ott SJ, Waetzig GH, Rehman A, Moltzau-Anderson J, Bharti R, Grasis JA, Cassidy L, Tholey A, Fickenscher H, Seegert D, Rosenstiel P, Schreiber S. 2017. Efficacy of sterile fecal filtrate transfer for treating patients with Clostridium difficile infection. Gastroenterology 152:799–811.e7. doi:10.1053/j.gastro.2016.11.010. 27866880

[8] Harcombe WR, Bull JJ. 2005. Impact of phages on two-species bacterial communities. Appl Environ Microbiol 71:5254–5259. doi:10.1128/AEM.71.9.5254-5259.2005. 16151111 PMC1214695

[9] Hsu BB, Gibson TE, Yeliseyev V, Liu Q, Lyon L, Bry L, Silver PA, Gerber GK. 2019. Dynamic modulation of the gut microbiota and metabolome by bacteriophages in a mouse model. Cell Host Microbe 25:803–814.e5. doi:10.1016/j.chom.2019.05.001. 31175044 PMC6579560

[10] Lourenço M, Chaffringeon L, Lamy-Besnier Q, Pédron T, Campagne P, Eberl C, Bérard M, Stecher B, Debarbieux L, De Sordi L. 2020. The spatial heterogeneity of the gut limits predation and fosters coexistence of bacteria and bacteriophages. Cell Host Microbe 28:390–401.e5. doi:10.1016/j.chom.2020.06.002. 32615090

[11] Breitbart M, Hewson I, Felts B, Mahaffy JM, Nulton J, Salamon P, Rohwer F. 2003. Metagenomic analyses of an uncultured viral community from human feces. J Bacteriol 185:6220–6223. doi:10.1128/JB.185.20.6220-6223.2003. 14526037 PMC225035

[12] Bondy-Denomy J, Davidson AR. 2014. When a virus is not a parasite: the beneficial effects of prophages on bacterial fitness. J Microbiol 52:235–242. doi:10.1007/s12275-014-4083-3. 24585054

[13] Hsu BB, Way JC, Silver PA. 2020. Stable neutralization of a virulence factor in bacteria using temperate phage in the mammalian gut. mSystems 5:e00013-20. doi:10.1128/mSystems.00013-20. 31992629 PMC6989128

[14] Hsu BB, Plant IN, Lyon L, Anastassacos FM, Way JC, Silver PA. 2020. In situ reprogramming of gut bacteria by oral delivery. Nat Commun 11:5030. doi:10.1038/s41467-020-18614-2. 33024097 PMC7538559

